# Safety planning and transdiagnostic cognitive behavioral therapy for adolescent suicide prevention in Mozambique: study protocol for the SPI/TCBT-S hybrid effectiveness/implementation cluster randomized trial

**DOI:** 10.1186/s12888-025-07102-w

**Published:** 2025-07-01

**Authors:** Bradley H. Wagenaar, Alberto Muanido, Morgan Turner, Kathryn L. Lovero, Doyanne Darnell, Monisha Sharma, Katherine Anne Comtois, Isaías Ramiro, Vasco F. J. Cumbe

**Affiliations:** 1 Department of Global Health, University of Washington, 3980 15Th Ave NE, Seattle, WA 98105 USA; 2 Department of Epidemiology, University of Washington, Seattle, WA USA; 3 Mozambique Health Committee, Beira, Mozambique; 4 Department of Sociomedical Sciences, Columbia University Mailman School of Public Health, New York, NY USA; 5 Department of Psychiatry and Behavioral Sciences, University of Washington, Seattle, WA USA; 6 Mozambique Health Committee, Maputo, Mozambique; 7 Provincial Health Directorate, Sofala Province, Ministry of Health, Beira, Mozambique; 8 Health Training and Research Center, Central Hospital, Beira, Mozambique

**Keywords:** Suicide prevention, Safety planning, Cognitive behavioral therapy, Cluster randomized trial, Adolescent health, Mozambique, Hybrid effectiveness/implementation trial

## Abstract

**Background:**

More than 75% of suicide deaths occur in low-and middle-income countries (LMICs) and almost 90% of adolescents who die by suicide live in LMICs. Globally, suicide is the fourth leading cause of death for youth aged 15–29. Six of the top 10 countries by suicide rates in the world are in the African region. Despite this, there are few to no evidence-based youth suicide prevention packages developed for, and tested in, the African context. To address this gap, this study aims to test effectiveness and implementation outcomes for the brief Safety Planning Intervention (SPI) and the more resource-intensive multi-session Transdiagnostic Cognitive Behavioral Therapy Intervention for Suicide Prevention (TCBT-S) delivered by non-specialists in Mozambican secondary schools.

**Methods:**

Using a three-arm, parallel, Hybrid Type-1 cluster randomized trial design, we will randomize 7 secondary schools each to Enhanced Usual Care (EUC), SPI alone, and TBCT-S (21 schools total) to evaluate effects on suicidal behaviors (primary) and suicidal ideation/depressive symptoms (secondary). Exploratory structural equation modeling will examine potential mechanisms of intervention effects. Implementation outcomes, barriers, and facilitators to EUC, SPI, and TCBT-S implementation will be assessed using the RE-AIM evaluation and CFIR determinant frameworks. Costs and cost-effectiveness will be evaluated using a Markov model parameterized with cost and trial outcomes data and be applied to projecting budget impact and potential scale-up to provincial and national levels.

**Discussion:**

This study is innovative in being the first, to our knowledge, to rigorously test SPI and TCBT-S for adolescent suicide prevention in Sub-Saharan Africa. By simultaneously testing the brief SPI alone – as well as integrated into a more resource-intensive TCBT-S – this study will examine whether the potential gains in effectiveness with the more resource intensive TCBT-S justify its scale-up versus the brief SPI intervention. If effective, SPI or TCBT-S have a large potential to be rapidly scaled up to safeguard youth mental health in Mozambique and other similar LMICs.

**Trial registration:**

ClinicalTrials.gov; NCT06465381; https://clinicaltrials.gov/study/NCT06465381.

**Supplementary Information:**

The online version contains supplementary material available at 10.1186/s12888-025-07102-w.

## Background

**Fig. 1:**
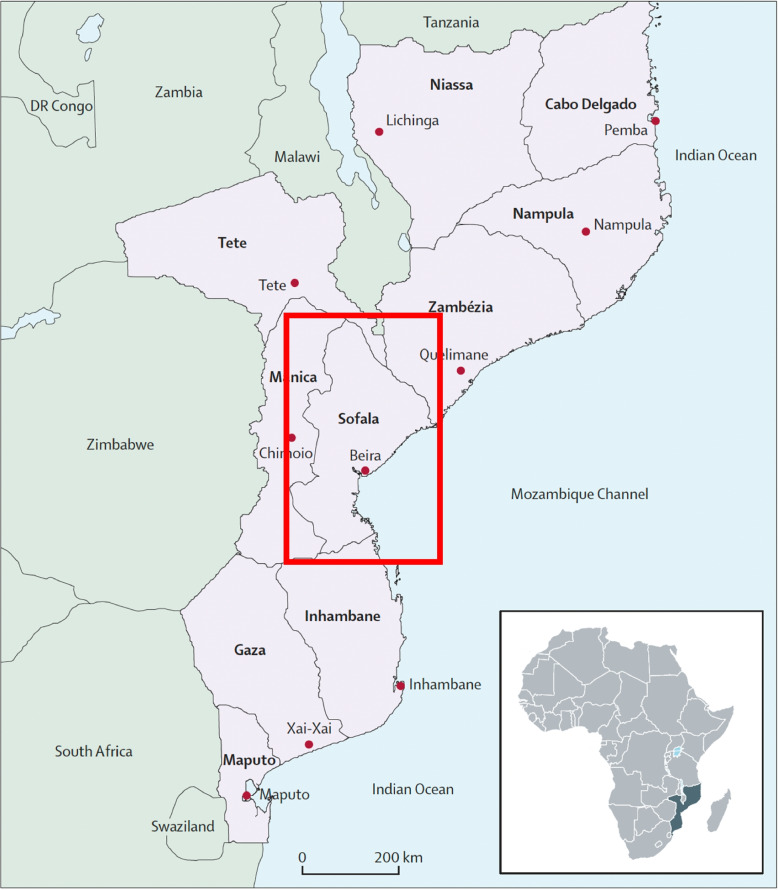
Mozambique with focal province of Sofala outlined in red. Figure sourced from Fernandes QF et al. (2014)

Youth suicide is a global public health emergency, with the African World Health Organization (WHO) region having the highest suicide rates in the world. More than three quarters of suicide deaths occur in low-and middle-income countries (LMICs) and almost 90% of adolescents who die by suicide live in LMICs [1] Globally, suicide is the fourth leading cause of death for adolescents and young adults aged 15–29 [2] The African WHO region has the highest estimated age-standardized suicide rates in the world, with countries like Mozambique (Fig. 1) having suicide rates almost three times the global average. Six of the top 10 countries by suicide rates globally are in the African region [2]. Meta-analyses have suggested that current rates of adolescent depression and anxiety are double pre-pandemic levels – worrying trends that may relate to even higher risks of suicidal behavior in this already at-risk group [3] These data have caused countries such as the US to deliver rare public health advisories about the urgent need to address youth mental health [4].

Limited research exists on youth suicide in the African WHO region and few to no evidence-based preventative interventions have been tested in LMICs more generally. On October 6th, 2022 the WHO launched a global campaign highlighting the urgent need to raise awareness and galvanize action for suicide prevention in the African region calling it a “crisis in Africa” [5]. Recent systematic reviews have shown that, while global suicide rates have fallen over the past decades, rates of suicide in LMICs continue to be high – especially for youth and young adults [6] – and in many African countries suicide rates are increasing [7]. Despite the high burden of suicide, LMICs and specifically the African region, are extremely underrepresented in research. A recent systematic review evaluating the effect of the COVID-19 pandemic on suicidal behavior noted a complete absence of any research from the African continent [8]. Systematic reviews of suicide prevention interventions in LMICs have shown a paucity of existing research on best evidence approaches, such as safety planning and support for individuals after suicide attempts [7]. A 2022 systematic review of suicide prevention studies among youth in LMICs [9] found only one small pilot study conducted in the African WHO region (South Africa) testing a brief suicide intervention among individuals living with HIV.

Suicide and suicidal behaviors are an urgent public health problem in Mozambique, with rates highest among adolescents and young adults, yet prevention interventions are unavailable. In a seminal 2013 study, our team showed that over 16% of suicide deaths from legal medicine autopsies were adolescents under the age of 18 and 48% of deaths were young people under the age of 25 [10]. Another representative community household study of 3,080 individuals by our team in Sofala and Manica Provinces showed that of the 17% of the sample reporting lifetime suicidal ideation, only 3.8% ever received any treatment for their suicidal thoughts [11]. We recently completed a study of adult suicidal behavior in primary care in Sofala Province showing strong associations with younger ages and high rates of suicidal plans; over 85% of those aged 18–24 with suicidal ideation had made a suicide plan and 65% of those with a suicide plan made a suicide attempt. These data suggest the urgent need to intervene in the prevention of suicide attempts specifically for younger individuals in Mozambique as the cascade from ideation to planning to attempt is very high in this context [12]. Last, a pilot study by our team among adolescents 12–19 in grades 8–12 in Mozambique secondary schools found 16% reporting past month suicidal thoughts, 11% reporting suicide planning, and 6% with past-month suicidal behavior; lifetime suicide attempts were also high at 9% of adolescents [13]. Despite these alarming statistics, no youth suicide prevention approaches have been tested or implemented in Mozambican schools.

Suicide safety planning and cognitive behavioral interventions to reduce suicide are best evidence interventions to reduce suicidal behavior in high-income contexts yet have never been tested in the African WHO region. A 2021 meta-analysis [14] of the effectiveness of suicide safety planning interventions showed very large effects on suicidal behavior (RR: 0.57; 95% CI: 0.41–0.79). Another more recent meta-analysis of the Safety Planning Intervention (SPI) found 26 studies globally [15], with only a single quasi-experimental study in a LMIC (India). This study found SPI to be feasible, acceptable, and flexible to various contexts. Meta-analyses of cognitive behavioral interventions to reduce suicide have also consistently shown very strong effect sizes in the reduction of suicidal behavior [16–19]. Yet, few studies have been conducted with adolescents and to our knowledge none of these systematic reviews include a single study testing cognitive behavioral interventions to reduce suicide in the African continent. In high-income settings, extensive evidence has been developed regarding SPI and cognitive behavioral interventions for the prevention of suicidal behavior, and in many contexts both interventions are being or have been scaled-up. Since 2016, the United States Joint Commission has recommended SPI as standard of care for suicide prevention [20]. By contrast, no high-quality evidence exists for the effectiveness of either SPI or cognitive behavioral interventions for suicide prevention in LMICs. This lack of evidence is directly hindering the ability to scale-up best evidence interventions to address the urgent need for suicide prevention in areas with the highest suicide rates globally [21, 22].

The Common Elements Treatment Approach—Mozambique (CETA-MZ) CBT-based transdiagnostic therapy is feasible, acceptable, and associated with decreases in depressive symptoms and suicidal ideation in Mozambique. Starting in 2018, our team led the adaptation of CETA to the Mozambican context. This included training/supervision of non-specialist healthcare workers and a demonstration project to test feasibility, acceptability, and clinical outcomes among patients testing HIV + in primary care clinics of Sofala, Mozambique. Initial data suggested that, among youth 18–24, CETA-MZ was associated with an 80% reduction in depression symptoms and a reduction in suicidal ideation from 20% at intake to < 3% after 5 sessions [21]. CETA and other cognitive behavioral interventions have also been tested in other contexts showing feasibility, acceptability, and effectiveness among youth < 18 and in schools [22–26].

We believe that these two evidence-based practices, both with demonstrated feasibility in Mozambique, have the potential to be powerful interventions to prevent adolescent suicidal behavior. Yet, a recent meta-analysis found lower comorbidity of psychiatric disorders and suicidal behavior in LMICs (~ 50%) compared to high-income countries (~ 90%) [27]. Therefore, it is possible that applying CETA-MZ to address psychiatric symptoms may not lead to significant decreases in suicidal behavior above and beyond SPI alone. For these reasons, we propose to test both SPI alone and the integration of SPI into CETA-MZ – adapted for adolescent suicide prevention – to create a Transdiagnostic Cognitive Behavioral Therapy for Suicide (TCBT-S) delivered by non-specialists in Mozambican secondary schools. Due to limited resources for mental health in the African region we aim to test whether the potential gains in effectiveness with the more resource intensive TCBT-S justify its scale-up versus the brief SPI intervention alone. This study will be conducted using a three-arm parallel Hybrid Type-1 cluster randomized trial design across 21 schools (7 to each arm of Enhanced Usual Care [EUC], SPI alone, and TCBT-S). This study will also generate evidence on costs, implementation determinants, and potential mechanisms of intervention effects to optimize intervention components and implementation strategies for future scale-up, if effective.

## Methods

### Safety Planning Intervention (SPI) specification and processes

**Fig. 2:**
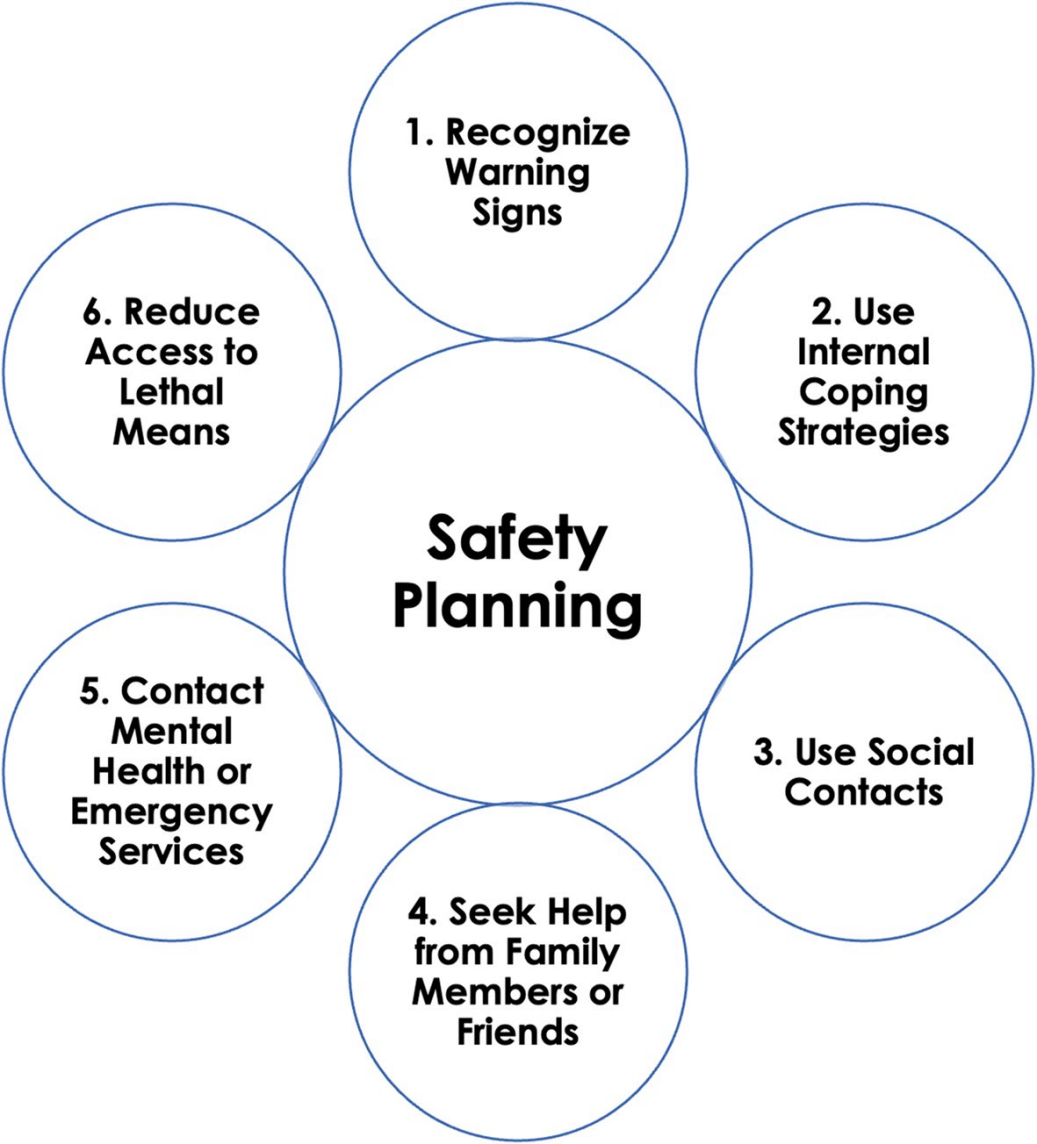
Schematic of the components of the Safety Planning Intervention (SPI)

SPI is an evidence-based brief intervention (~ 30–45 min) that provides strategies to decrease the risk of suicidal behavior (Fig. 2). It has been shown to be effective at reducing suicidal behavior in adults and adolescents in high-income countries [28–30] and is a best practice for the Suicide Prevention Resource Center/American Foundation for Suicide Prevention [30]. SPI begins with psychoeducation about suicide risk followed by a collaborative approach to create a stepwise safety plan that includes a list of individually tailored, concrete coping mechanisms to be enacted during or leading up to a crisis. The six steps of the safety plan include: 1) recognizing warning signs of a crisis; 2) employing internal coping strategies; 3) using social contacts and settings to distract from suicidal thoughts; 4) seeking help from family members or close friends; 5) contacting healthcare or emergency services; and 6) reducing access to means [28, 31].

### Transdiagnostic CBT-based intervention for Youth Suicide Prevention (TCBT-S) specification and processes

**Fig. 3:**
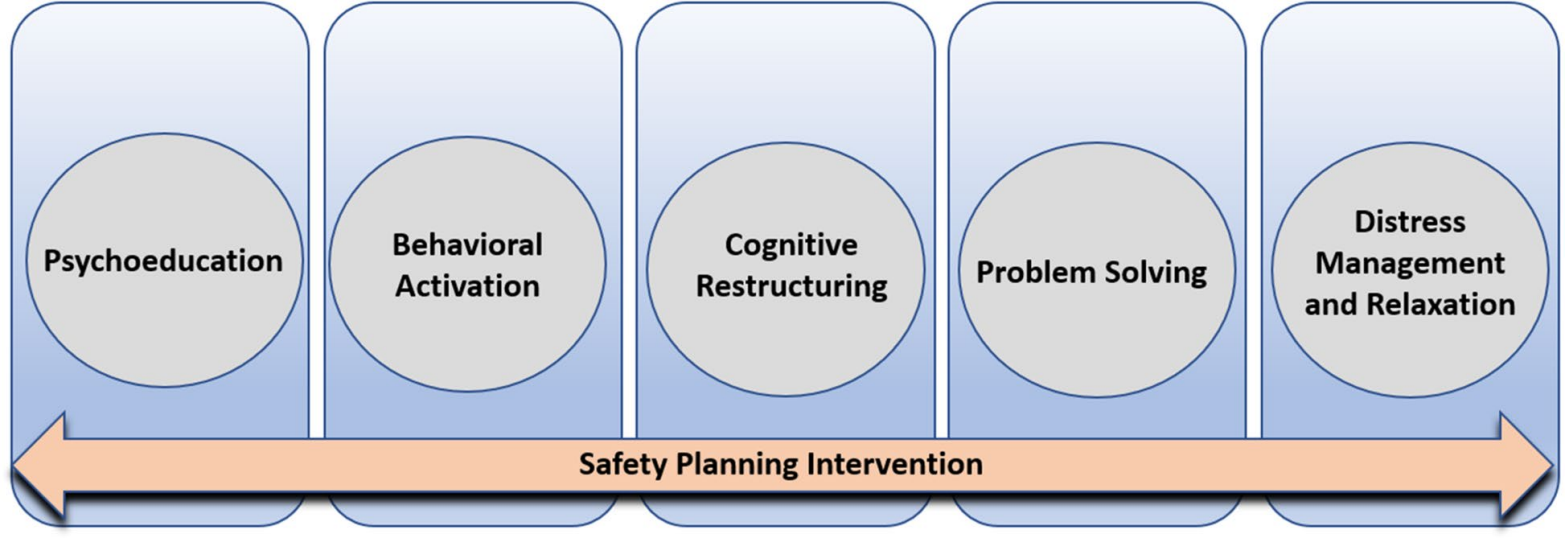
Schematic of the components of the Transdiagnostic CBT-based Intervention for Youth Suicide Prevention (TCBT-S)

CETA-MZ is an evidence-based, manualized transdiagnostic CBT-based protocol to address co-morbid common mental disorders [32]. CETA-MZ leverages the commonalities in CBT approaches to treatment of depression, anxiety, and PTSD in a single flexible intervention for one or more of these often-overlapping disorders. CETA-MZ is a Mozambique adaptation of CETA that has been adopted by the Ministry of Health for national scale-up in HIV/AIDS settings [21, 33]. The present study will optimize CETA-MZ clinical protocols to specifically address youth suicide prevention as well as integrate the full SPI intervention simultaneously to create a novel Transdiagnostic CBT-based Intervention for Youth Suicide (TCBT-S); (Fig. 3) [34]. We will retain individual modules: (1) Psychoeducation (CETA module “Introduction/Encouraging Participation”); (2) Behavioral Activation (“Getting Active”); (3) Cognitive Restructuring (“Thinking in a Different Way Part 1 & 2”); (4) Problem Solving (“Resolving Problems”); and (5) Distress Management and Relaxation Strategies (“Relaxation”). SPI will be embedded in TCBT-S, including creation of the safety plan at first contact, and revisiting the plan at following visits to make plan adjustments, if needed [34]. Adaptations to CETA-MZ modules will include: i. psychoeducation focused on suicidal thoughts and behaviors and how TCBT-S is expected to contribute to recovery; ii. cognitive restructuring adapted to focus on the thoughts and feelings that occur during, or leading up to, suicidal thoughts or behaviors and techniques for evaluating and reframing firmly held thoughts that can contribute to suicidal crisis; iii. linking behavioral activation to coping strategies included in the safety plan for acute distress tolerance and crisis avoidance; iv. problem solving focused on addressing problems believed to be contributing to suicidal thoughts and behaviors; and v. distress management and relaxation strategies specifically targeted towards emotional regulation to manage overwhelming physical and emotional response contributing to suicidal thoughts and behaviors.

### Study aims

In this proposal, we will conduct a Hybrid Type 1 effectiveness/implementation study, following the typology outlined by Curran et al. [35, 36]. In a Hybrid Type 1 study, the primary study aim is to test clinical intervention effectiveness paired with a secondary aim to better understand implementation outcomes and the implementation context. Type 1 trials are best in situations where an intervention has strong face validity and indirect evidence for applicability to the population in question which we argue hold given extensive evidence for effectiveness of SPI and CBT interventions for suicide prevention in high-income contexts. We use the SPIRIT guidelines to structure this study protocol [34].

The present project proposes to conduct a 2-year parallel Hybrid Type-1 cluster RCT, randomizing 21 government secondary schools to enhanced usual care (EUC), Safety Planning Intervention (SPI) alone, and the Transdiagnostic CBT-based Intervention for Youth Suicide (TCBT-S); (Table 1). Primary study outcomes will be evaluated at 6 months post intake, with maintenance indicators at 12 and 24 months. A sub-aim of the cluster RCT will be an exploratory evaluation of causal pathway models to examine mechanisms of action of SPI and TCBT-S interventions using longitudinal structural equation modeling.

Alongside testing intervention effectiveness, we will unpack implementation context and potential problems associated with real-world implementation by linking quantitative implementation outcomes based on the RE-AIM evaluation [37] with determinants (barriers/facilitators) based on the Consolidated Framework for Implementation Research (CFIR) [38, 39]. Last, we will estimate the incremental cost and cost-effectiveness of scaling-up SPI and TCBT-S in Mozambique using micro-costing, time-and-motion observation, and a Markov model parameterized with cost and outcome data from the SPI and TCBT-S cluster RCT.

**Table 1:**
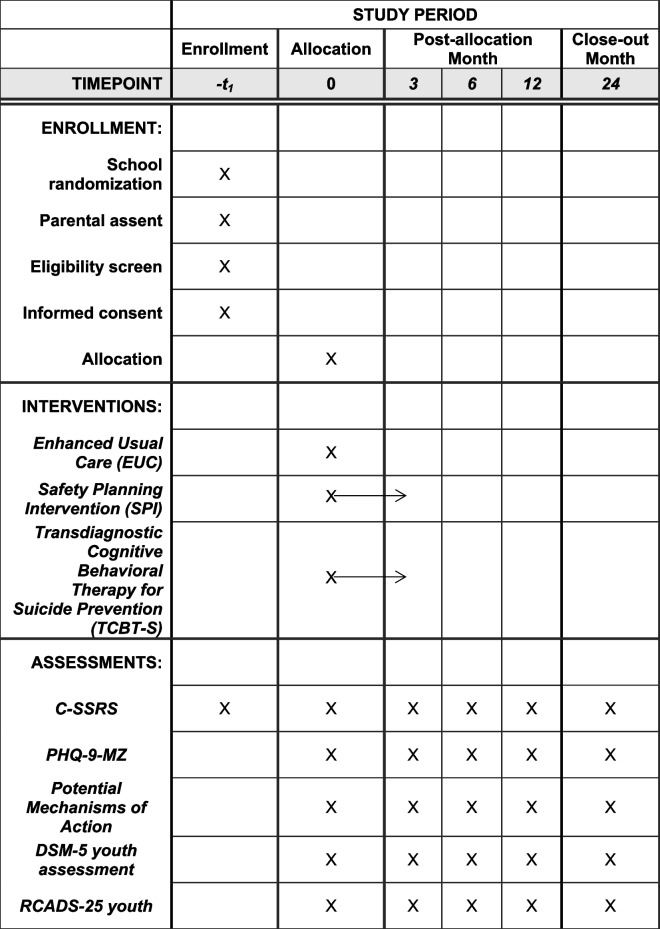
SPIRIT project activities and timeline

### Overview of study design

Following IRB approvals, planning meetings, and randomization of secondary schools, our team will recruit non-specialist healthcare workers and train them to lead screening activities using the Columbia Suicide Severity Rating Scale (C-SSRS) for youth [40, 41] as well as EUC, SPI, or TCBT-S treatment protocols (see Table 1 for full study timeline). The C-SSRS defines clear levels of suicide risk and guidance for their use in clinical trials that have been adopted by the FDA [42]. Youth enrolled in grade 10 at target schools (Table 2) will be screened for active suicidal ideation in the past month and those who screen positive (responding affirmatively to “have you actually had any thoughts about killing yourself?” on the C-SSRS) will be enrolled in EUC, SPI, or TCBT-S respectively until recruitment targets are met. EUC protocols will apply to all arms of the study and include identification of suicide risk through the C-SSRS. Those determined to be high risk using the C-SSRS will be transported to local government emergency mental health services that Dr. Cumbe (Co-I) directs in his role as Provincial Director of Mental Health services in Sofala Province. All selected schools have nearby emergency psychiatric and psychological services (< 30 min); (Table 2). These activities will include collaboration with Youth Friendly Services at each health facility. EUC activities will be implemented by nonspecialist healthcare workers who will participate in a 3-day practical training provided by Mozambican SPI/TCBT-S trainers supervised by Drs. Darnell, Lovero, and Cumbe. For students with moderate risk, they will receive information and referral to local government mental health and youth friendly services. At follow-up outcome ascertainment timepoints, those in the EUC arm will be re-assessed for suicide risk and uptake of referral services.

**Table 2:** Target schools and characteristics

N	School	# Enrolled in 10th Grade, 2024	Referral Health Facility	District	Urban, Rural, or Peri-urban
1	Escola Secundária da Ponta Gea	542	CS Ponta Gêa	Beira	Urban
2	Escola Secundária Samora Moises Machel	1134	CS da Ponta Gêa	Beira	Urban
3	Escola Secundária Mateus Sansão Mutemba	1674	CS da Ponta	Beira	Urban
4	Escola Secundária da Manga	1433	CS de Nhaconjo	Beira	Urban
5	Escola Secundária do Estoril—Macuti	1051	CS de Macurungo	Beira	Urban
6	Escola Secundária 25 de Setembro—Chamba	594	CS de Inhamizua	Beira	Peri-urban
7	Escola Secundária de Marocanhe	864	CS Mascarenhas	Beira	Peri-urban
8	Escola Secundária de Matadouro	1131	CS de Inhamizua	Beira	Peri-urban
9	Escola Secundária de Muchatazina	860	CS da Munhava	Beira	Urban
10	Escola Secundária do Dondo	1105	CS de Dondo	Dondo	Urban
11	Escola Secundária de Mafambisse	926	CS de Mafambisse	Dondo	Rural
12	Escola Secundária de Macharote	840	CS de Dondo	Dondo	Peri-urban
13	Escola Secundária Graça Machel-Dondo	395	CS de Dondo	Dondo	Rural
14	Escola Secundária Filipe Samuel Magaia	305	CS de Mafambisse	Dondo	Rural
15	Escola Secundária de Nhamatanda	729	HR de Nhamatanda	Nhamatanda	Peri-urban
16	Escola Secundária de Lamego	244	HR de Nhamatanda	Nhamatanda	Rural
17	Escola Secundária de Metuchira	445	HR de Nhamatanda	Nhamatanda	Rural
18	Escola Secundária Marcelino Dos Santos	630	HR de Nhamatanda	Nhamatanda	Peri-urban
19	Escola Secundária Mathias Manuel Kaphesse	713	HD de Caia	Caia	Peri-urban
20	Escola Secundária de Inhaminga	542	CS de Inhaminga	Cheringoma	Rural
21	Escola Secundária de Muxúngue	275	HR de Muxúngue	Chibabava	Rural

In the SPI only arm, following previously tested and piloted protocols in Mozambique, non-specialist healthcare workers will participate in a 5-day practical training provided by Mozambican SPI trainers. Following the apprenticeship model [43], SPI providers will role-play suicide screening and SPI protocol implementation in weekly supervision meetings with trainers. Once providers show competence in role-play they will be certified using the Safety Plan Intervention Rating Scale for Mozambique (SPIRS-MZ) [44] where they will have to achieve adequate or better rating on each scale domain. Consistent with expert recommendations on SPI [28, 31], youth will receive follow-up contacts at two and four weeks post intake to assess risk and review their safety plan. Safety plans will also be reviewed during outcome measurements at 3, 6, 12, and 24 months.

For the TCBT-S arm, non-specialist healthcare workers will be trained in the more complex CBT-based intervention. Following successful CETA-MZ protocols [21, 32, 43], this includes 2 weeks of practical training, followed by a minimum of one month of supervised TCBT-S practice and role-plays. Providers will only be certified once they show full competency in delivering TCBT-S sessions per protocol. As is done in CETA-MZ implementation, prior to implementation of each element with each student (e.g. cognitive restructuring), TCBT-S providers will discuss each case with TCBT-S supervisors and must show competency in the element prior to engaging in the session. TCBT-S will be implemented in 6–8 sessions spaced one week apart. Treatment completion will be defined as completing all required TCBT-S sessions and showing competency and understanding in the skills as demonstrated through homework and in-session exercises. Given the complexity of some sessions – particularly cognitive restructuring – some youth may take more than six sessions to show competency. For youth in the TCBT-S arm, they will construct their safety plan at intake paired with the first session of TCBT-S including an introduction to the program and psychoeducation focused specifically on suicidal thoughts and behaviors and how TCBT-S is expected to contribute to recovery. For example, some may need to repeat elements multiple times prior to understanding and demonstrating competency [45–47].

### Study setting

Sofala Province (population ~ 2.4 million; Fig. 1) was selected because of the deep relationship between investigators and the local MoH as well as the absence of existing interventions for youth suicide prevention. While only approximately 40–50% of youth in Mozambique attend secondary school [45], the decision to implement the present study in secondary schools in Sofala, Mozambique was based on: (1) our previous challenges with longitudinal treatment of youth in primary care settings; (2) feasibility of recruiting a large cohort of youth with active suicidal ideation as well as treatment stigma in community settings; and (3) preliminary studies documenting high rates of suicidal behavior among secondary students. If effective, we see potential for SPI or TCBT-S to be scaled-out and implemented in other community-based healthcare settings, such as youth friendly health services, emergency rooms, or primary care settings for larger population reach. In Mozambique, over 98% of formal health services are offered through the Ministry of Health [46], improving the potential for rapid national scale-up when collaborating and implementing within the centralized government systems. This has been the case with the original CETA-MZ intervention, which is currently being scaled-up nationwide in HIV/AIDS care settings.

### Study outcomes

Primary study outcomes will be youth-level suicidal behavior in the past month (primary), suicidal ideation in the past month (secondary), depressive symptoms in the past 2 weeks (secondary); (Table 3). Suicidal behavior was selected as the primary trial outcome due to previous meta-analyses showing SPI and CBT interventions for suicide prevention to have the strongest effects on reducing suicidal behavior [14, 17]. In the present trial, all primary and secondary outcomes will be collected at 3-, 6-, 12-, and 24-months post intake. Primary analyses will occur at 6 months following the strongest evidence from previous trials. Secondary outcomes of suicidal ideation and depressive symptoms are included due to mixed evidence on these outcomes from CBT-based suicide prevention meta-analyses [16, 17]. The DSM-5 crosscutting assessment and the RCADS-25 youth will also be collected at all timepoints due to requirements imposed by the NIMH Common Data Elements [47]. As shown in Table 3, suicidal behavior will be measured through reported actual suicide attempts, interrupted attempts, aborted attempts, or specific preparatory acts or behavior reported on the C-SSRS for youth [40, 41]. Our team has previously translated, adapted, and applied the C-SSRS to evaluate suicidal behavior among youth in schools in Mozambique and the instrument was acceptable, appropriate, and feasible to implement [13]. Suicidal ideation will include active suicidal thoughts reported in the past month on the C-SSRS. Clinically-significant depressive symptoms will be measured through the PHQ-9-MZ, a Mozambique adaptation of the PHQ-9 that our team has previously validated for use in Sofala, Mozambique [48]. Patient-level data collection will be done in CommCare [49], which our team is currently using for longitudinal patient tracking for a mental health cRCT in Sofala Mozambique [47]. All data will be stored on encrypted and password protected computers, accessible only to the named study staff. Any identifying features needed to link follow-up of patients over time will be destroyed at the end of the 24-month follow-up period.

**Table 3:** Primary and secondary clinical outcome indicators and definitions

Outcome	Indicator	Definition
PRIMARY	Suicidal behavior in past month	Actual suicide attempt, interrupted attempt, aborted attempt, or specific preparatory acts or behavior reported on the C-SSRS in past month
SECONDARY	Suicidal ideation in past month	Non-specific active suicidal thoughts reported on C-SSRS in past month
SECONDARY	Depressive symptoms in past two weeks	PHQ-9-MZ score ≥ 10 referring to symptoms in the past 2 weeks

### Sampling and randomization procedures

Eligible schools are public-sector government secondary schools located in Sofala Province, Mozambique and with a < 30-min drive from an emergency department with co-located government psychiatric/mental health services. These 21 target schools had a total of 16,432 students enrolled in normal daytime classes of 10th grade in 2024 (Table 2). We will randomly allocate the 21 schools 1:1:1 to the three arms using constrained randomization to balance school size (number of total students enrolled in 10th grade in 2024) and rural/peri-urban/urban location. In previous work among youth in schools in Mozambique we had approximately 90% of enrolled students return parental consent forms and participate [13]. Randomization will be done using the cvcrand command in Stata. Thus, we anticipate screening approximately 14,788 students for active sucidal ideation in this trial. Based on pilot data [13] we estimate approximately 14% of those screened to express active suicidal ideation. Recruitment will progress until 2,100 youth are recruited and enrolled in the study across arms. If needed to achieve target recruitment, we will recruit additional students balanced across arms attending 9th or 11th grades.

### Primary statistical analyses

Individual-level primary and secondary outcome data will be analyzed using generalized linear mixed models using the binomial family, logit link, and random effects at the school and individual level. Primary analyses will compute odds ratios for suicidal behavior in the past month comparing the SPI alone and the TCBT-S arms to EUC; primary outcome analyses will be done at 6 months post intake. We will also conduct supplementary analyses of primary and secondary outcomes at 3 months post intake. Sensitivity analyses will utilize gaussian models to directly compute differences in the proportion of SPI and TCBT-S with suicidal behavior compared to EUC. Student (age, sex, grade) and school (size, location) will be considered for inclusion based on balance. Secondary analyses will utilize the same modeling approaches to evaluate odds ratios for suicidal ideation and PHQ-9 ≥ 10 as well as directly comparing outcomes between SPI alone and TCBT-S arms. We will also examine absolute differences in PHQ-9-MZ scores across groups using gaussian models. Sex as a biological variable will be assessed using pre-specified interaction sub-analyses to examine differential SPI and TCBT-S effects for females vs. males. For the RE-AIM maintenance domain, we will repeat all analyses at 12 and 24 months. All statistical analyses will be conducted blinded to study arm.

### Primary study power assessment

A 2021 meta-analysis of 6 studies of suicide safety planning interventions in high-income contexts found a global relative decrease in SB of 43% (RR: 0.57; 95% CI: 0.41–0.79). Using a conservative alpha value of 0.025 to account for multiple comparisons in the 3-arm cRCT design, an ICC of 0.02, estimated average cluster size of 100 students expressing active SI (14% of 14,788 students screened), we will have > 80% power for a conservative relative decrease in SB of 35%. This represents an absolute difference of 13%, with an estimated 37% of control students with SB in the past month compared to 24% in the intervention groups. See Table 4 for power under varying effect estimates and mean number of students with active SI. Currently there is no consensus in the statistical literature on whether three-arm cRCTs should adjust their alpha value for multiple comparisons with a concurrent control group; half of published trials have adjusted for multiple comparisons and half have not adjusted [50, 51]. Since our two arms are testing a related treatment (SPI alone versus TCBT-S) we argue that more stringent control of family-wise error rate should be employed. Thus, we have chosen to use the conservative Bonferroni correction to account for multiple comparisons in our primary outcome assessment (Alpha = 0.05/N primary outcome tests = 0.025). If we employ an alpha of 0.05, we will have > 80% power to detect a relative decrease of 32.5% (an absolute difference of 12%).

**Table 4:** Power under various conditions. Green indicates power > 80%

Mean number of students with active SI per school						
**Primary Outcome:** Decrease in Suicidal behavior in past month from 37% (p1-p0)		60	80	100	120	140
	12.5%	.67	.72	.76	.79	.81
	15%	.84	.89	.92	.93	.94
	17.5%	.95	.97	.98	.99	.99
	20%	.98	>.99	>.99	>.99	>.99

### Assessment of implementation outcomes and barriers/facilitators to EUC, SPI, and TCBT-S implementation using the RE-AIM evaluation framework

We will embed a strong evaluation of the implementation context for the SPI and TCBT-S interventions. We will utilize two dominant frameworks in implementation science: (1) the RE-AIM evaluation framework [37] to examine implementation outcomes that impact the potential population-health impact of the SPI and TCBT-S interventions; and (2) the Consolidated Framework for Implementation Research (CFIR) determinant framework [38, 39] to systematically assess barriers and facilitators to implementation of the SPI and TCBT-S interventions. Our overall approach will follow a sequential QUAN- > qual explanatory analyses [52] whereby the CFIR framework will be used to help understand results found through our RE-AIM analyses. For example, if we find higher drop-out rates in one arm versus another or find specific schools or individuals with exceptionally positive or negative outcomes we can utilize CFIR focus groups and in-depth interviews to help explain determinants of these outcomes. Together, this rigorous evaluation aims to provide qualitative explanatory contextual models for differences in quantitative implementation outcomes across schools and groups of students. By identifying these barriers and facilitators to implementation, this aim will inform implementation and scale-up strategies for SPI and TCBT-S in Mozambique and other LMICs.

**Fig. 4:**
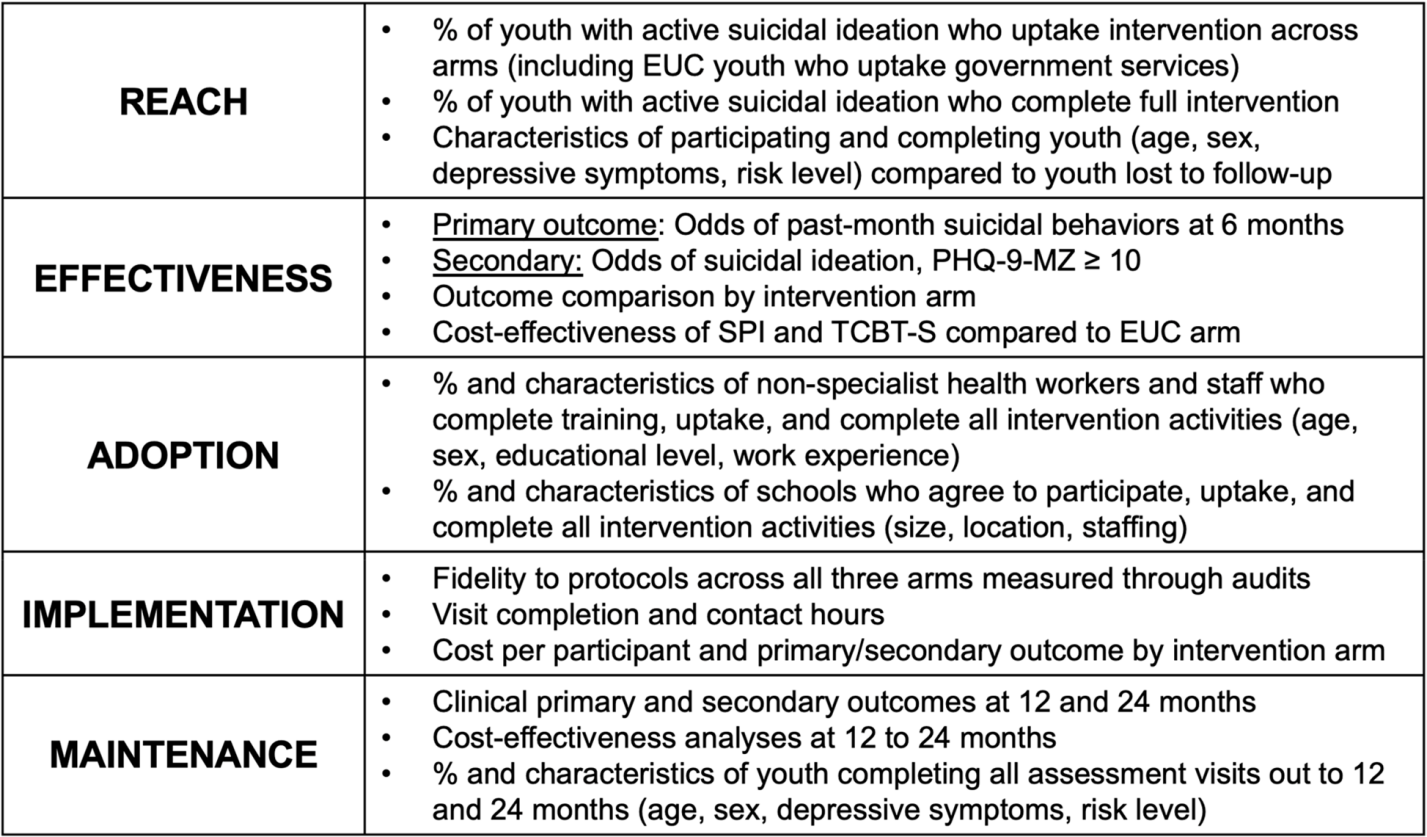
Proposed RE-AIM indicators for implementation outcome evaluation

RE-AIM implementation outcomes will be collected throughout trial implementation, summarized at the cluster (school) level, and examined separately across the EUC, SPI alone, and TCBT-S arms; (see Fig. 4). Student-level data will be collected electronically in CommCare [49] alongside primary outcome data during enrollment and intervention administration [47].

Reach analyses will describe youth participation and completion rates and the characteristics of youth reached compared to those not reached. We will utilize sociodemographic characteristics of the planned Mozambique Demographic and Health Survey (2022/3) [53] to compare the representativeness of enrolled youth to the overall population of youth in Sofala Province. We will also examine sociodemographic characteristics of youth who complete intervention activities versus those who drop out or are lost to follow-up.

Effectiveness endpoints will be assessed as described in study outcomes and primary statistical analyses sections.

Adoption will examine the proportion and characteristics of non-specialized health workers and staff who complete training and implement trial activities with at least one youth across arms as well as the proportion and characteristics of schools who agree to participate, uptake, and complete all intervention activities.

Implementation characteristics will include fidelity assessment across the three arms, overall visit completion and contact hours between implementation staff and youth in schools, and cost per participant and primary and secondary outcome by arm.

Fidelity for the SPI arm will be measured using the Safety Plan Intervention Rating Scale for Mozambique (SPIRS-MZ). The SPIRS scale [44] has been used in various contexts, including Mozambique, to certify providers after training in SPI and to monitor fidelity to core intervention elements. SPI fidelity will be assessed by Drs. Cumbe and Lovero through a sample of audio recordings of non-specialist workers administering the intervention. Fidelity monitoring for TCBT-S will include methods successfully used in previous CETA and CBT-based RCTs and following expert approaches for fidelity monitoring in community-based settings [54]. Both provider adherence (completion of required treatment components) and competence (degree of skill) in delivering TCBT-S will be monitored and systematically rated by Dr. Cumbe. Dr. Cumbe will review documentation of sessions to monitor adherence and observe a subset of recorded TCBT-S sessions representing the full range of clinical elements to rate clinical adherence and competence. As is routine during CETA-MZ implementation, Dr. Cumbe will review cases with providers and provide feedback on protocol adherence and competence during weekly supervision. Low adherence or competence will result in additional supervision and training for both SPI and TCBT-S. Fidelity for the EUC arm will include detailed monitoring of appropriate referrals to routine government services (emergency, psychiatric, psychological).

Maintenance will include assessment of extended clinical effects on primary and secondary outcomes through re-administration of assessment tools at 12- and 24-months post enrollment as well as proportion and characteristics of youth retained out to 12 and 24 months.

### Qualitative data collection and analyses

Following quantitative RE-AIM analyses, we will next conduct an explanatory qualitative analysis to determine the innovation, individual, inner setting, outer setting, and processes that distinguish between high- and low-performing schools and youth in each arm. These analyses will be guided by selected CFIR constructs in each domain chosen based on preliminary implementation experience with SPI and CETA-MZ in Mozambique (Table 5) although additional constructs may be included as novel relevant findings emerge throughout the project. Some constructs such as structural characteristics and cost will be covered through quantitative data collection [55]. To conduct these analyses, after we have 6 month primary outcome data, schools in each arm will be rank-ordered on magnitude of clinical effectiveness and we will select the 2 highest and 2 lowest ranking clusters in each arm (12 schools total). We will also examine other RE-AIM indicator performance (with the exception of maintenance) prior to selection to consider inclusion of additional clusters based on those indicators (e.g. clusters with very high or very low participation rates/visit completion). In selected clusters we will also rank-order and examine effectiveness and participation rates of individual students.

**Table 5:** Constructs of interest from the Consolidated Framework for Implementation Research (CFIR)

**I. INNOVATION**	
Innovation Source	1
Evidence Base	1
Relative Advantage	1
Adaptability	1
Trialability	1
Complexity	1
Design	1
Cost	2
**II. OUTER SETTING**	
Critical Incidents	1
Local Attitudes	1
Local Conditions	1
External Pressure	1
**III. INNER SETTING**	
Structural Characteristics	2
Culture	1
Compatibility	1
Mission Alignment	1
Available Resources	1
Access to Knowledge	1
**IV. INDIVIDUALS**	
Opinion Leaders	1
Innovation Deliverers	1
Innovation Recipients	1
High/Mid-level Leaders	1
Implementation Team	1
**V. PROCESS**	
Assessing Needs	1
Assessing Context	1
Tailoring Strategies	1
Engaging	1
Adapting	1

1 = qualitative data collection

2 = quantitative data collection

Subsequently, we will conduct 12 focus group discussions (FGDs) among high- and low-performing schools (anticipated 5–10 youth per FGD) and an anticipated 15 in-depth interviews (IDIs) with individual high or low-responding youth. These IDIs will allow for exploration of more sensitive topics and individual perceptions not easily expressed by youth in FGDs. In addition, IDIs will be informed by key themes emerging during FGDs and collect additional target information on emerging themes. If FGDs are deemed not feasible due to sensitivity of youth discussing suicide-related behavior in group format, we will conduct all IDIs. We will also conduct IDIs with a subset of non-specialist healthcare workers supporting the implementation of EUC, SPI, or TCBT-S (anticipated 15 workers). Last, at the end of the final maintenance phase (24 months post enrollment) we will conduct an additional 15 IDIs with youth to examine determinants of exemplary clinical sustainment or activation of key mechanisms.

IDIs and FGDs will be conducted in Portuguese by an experienced facilitator accompanied by a note-taker, audio-recorded, and transcribed verbatim into Portuguese by trained Mozambican staff. Using existing CFIR codebooks as a guide [55], two researchers will use a stepwise, iterative process to review transcripts and identify key deductive themes (using select CFIR constructs, RE-AIM outcomes) while allowing for flexibility of emergent inductive themes. Following iterative coding of transcripts, the trained Mozambican coding team will convene with the PI (BHW) to identify coding discrepancies and assess inter-coder agreement [56, 57]. Once consensus has been reached, two researchers will code all transcripts, and final measures of inter-coder agreement will be conducted (Cohen’s kappa) [58]. If final kappa measurements fall below 0.80, additional reviews of codes will be conducted, and the process will be repeated until agreement is achieved.

### SPI/TCBT-S mechanisms assessment approach

**Fig. 5:**
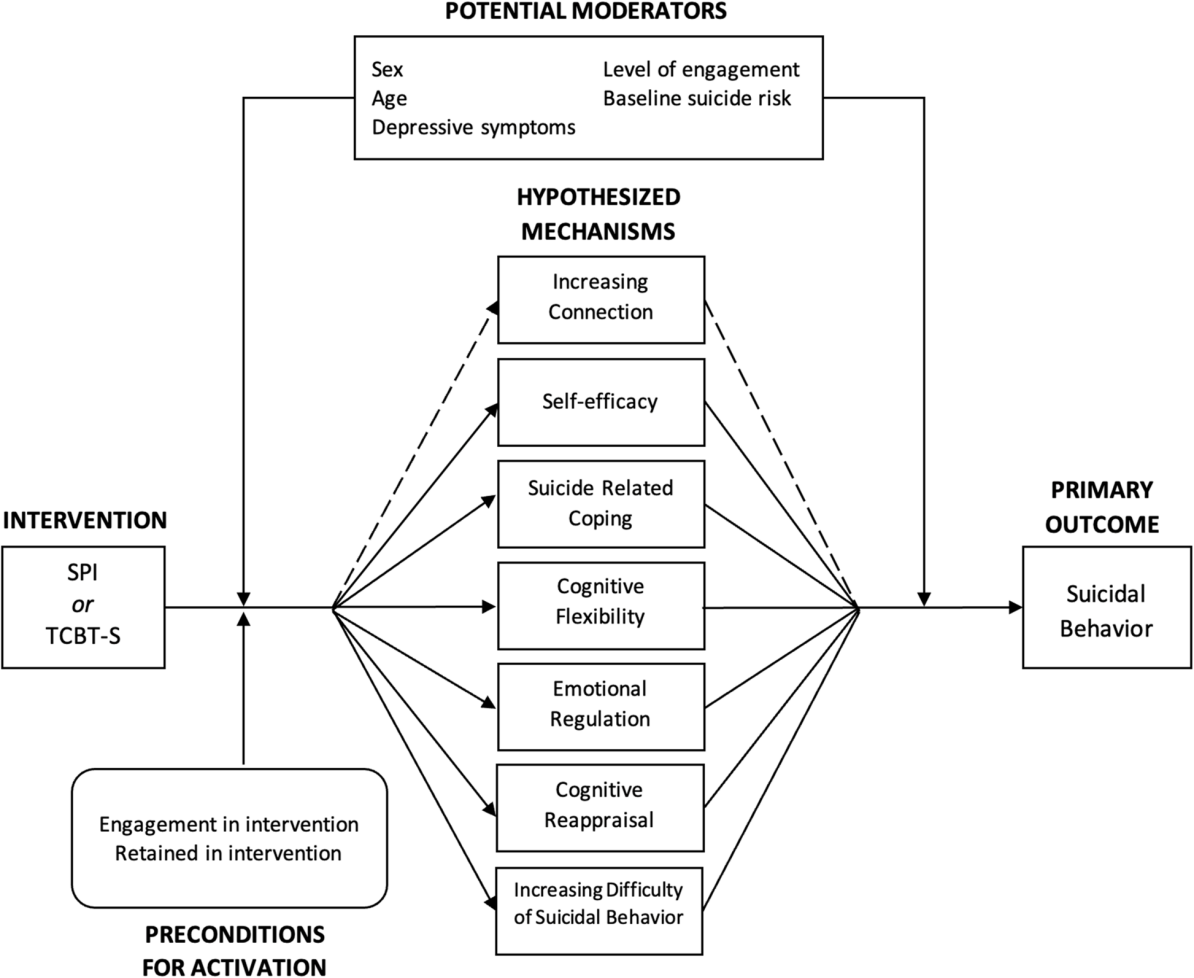
Causal pathway model of hypothesized mechanisms of SPI and TCBT-S effects

Building on existing published theoretical models of SPI and TCBT-S [30, 59], ongoing studies examining mechanisms of effects [60–62], as well as recent reviews [63] proposing mechanisms of action for SPI and cognitive behavioral interventions for suicide prevention [64] we have specified an intervention-mechanism-outcome conceptual model including six hypothesized mechanisms of action for effects of SPI alone and TCBT-S (see Fig. 5). Utilizing mechanism outcome data collected at intake and longitudinally we will test effects of hypothesized mechanisms and moderators using longitudinal structural equation modeling. Hypothesized mechanisms, preconditions, and moderators will be collected at intake and each effectiveness outcome measurement (3, 6, 12, and 24 months). Table 6 summarizes use of validated measurement approaches [65–70] (where available) to be used for each mechanism. To facilitate feasibility of measurement across numerous mechanisms scales, our team adapted questions from otherwise validated measures (Table 6), selecting questions with high factor loadings from applicable constructs. Primary analyses of mechanisms will occur at the end of the primary effectiveness outcome (6 months). Secondary analyses will occur at the end of the maintenance phase (24 months).

**Table 6:** Variable definitions for mechanistic structural equation model

Construct	Measurement Approach
***Moderators***	
Patient engagement	Study Visit Reports
Sex	Study Enrollment Form
Age	Study Enrollment Form
Baseline Suicide Risk	Columbia Suicide Severity Rating Scale (C-SSRS)
Depressive Symptoms	Patient Health Questionnaire (PHQ-9-MZ)
***Mechanisms***	
Cognitive Reappraisal	Emotion Regulation Questionnaire for Children and Adolescents (ERQ-CA)
Cognitive Flexibility	Cognitive Flexibility Inventory (CFI)
Suicide Related Coping	Suicide Related Coping Scale (SRCS)
Increasing Connection	EPOCH Measure of Adolescent Well-Being, connectedness subscale
Self-efficacy	General Self-efficacy Scale (GSES)
Emotional Regulation	The Difficulties in Emotion Regulation Scale—Short Form (DERS-SF)
Increasing Difficulty of Suicidal Behavior	Study specific questionnaire

### SPI/TCBT-S mechanisms assessment power

Overall, this study was powered to test the effectiveness of SPI or TCBT-S to decrease odds of suicidal behavior in the past month. Given the lack of previous research on mechanisms for SPI and CBT-based interventions for suicide prevention in the African context, this aim will focus on an exhaustive examination in search of practically- and clinically meaningful mechanism and moderator effect estimates for further examination in subsequent research. The goal of this aim is to contribute to theory building to inform future studies specifically powered to test hypothesized causal pathway models. No confirmatory hypothesis testing driven by p-value inference will be performed, as recommended when conducting exploratory studies by Leon (2011) [71]. However, we will have a sufficient sample size to have stable effect estimates with reasonable precision based on our design including a baseline measurement and 4 follow-up measurements among a minimum of 100 youth across 21 schools (10,500 observations total).

### SPI/TCBT-S mechanisms assessment analyses

Our analytical plan will progress in 3 stages. First, we will conduct univariate examination of hypothesized mechanism effects using multi-level longitudinal SEM accounting for multiple students nested within schools across time. The purpose of this will be to examine single main effect estimates for hypothesized mechanisms. For example, we will estimate a univariate multi-level longitudinal SEM of both SPI and TCBT-S intervention effects predicting increased social connectedness then increased connectedness predicting decreased suicidal behavior (dashed line in Fig. 5). We will conduct diagnostics, including assessing linearity of mechanism effects, model residual distributions, and variance accounted for in mechanisms and outcomes. We will then test potential moderators in univariate models, in search of those that strongly affect strength or direction of univariate effects. Moderators will be examined for their effect on intervention activation of mechanisms (left side of Fig. 5) as well as mechanism effects on suicidal behavior (right side of Fig. 5). Second, we will include all mechanisms simultaneously in a multivariable longitudinal multi-level SEM. Including all mechanisms, even those showing no effects in univariate models accounts for potential suppressor effects [72]. Moderators that showed meaningful effects in univariate models will then be included in a final multivariable model. Final model diagnostics will be verified, and variance accounted for in mechanisms, as well as outcomes, will be reported. This multivariable model will allow examination of: (1) mechanisms most affected by SPI and TCBT-S interventions (left side Fig. 5); (2) mechanisms that influenced outcomes (right side Fig. 5); and (3) the product of #1 and #2 path coefficients indicating the indirect effect of SPI or TCBT-S on treatment outcomes operating through a given mechanism. Separating paths #1 and #2 can inform intervention improvements; for example, if a mechanism had a large effect on outcomes (path #2) but was only weakly activated by the SPI intervention (path #1), the intervention could be modified to enhance activation of this potentially high-leverage mechanism.

### Costs

We will conduct activity-based costing and time and motion observation in all three study arms to estimate the incremental cost of implementing EUC, SPI, and TCBT-S. Throughout study implementation, we will estimate all costs that would be incurred by the Ministry of Health to administer the EUC, SPI, and TCBT-S interventions. These costs will be collected and tabulated using activity-based cost menus during start-up and trial implementation. Costs include screening, youth counseling sessions, emergency referrals, personnel, supervision, supplies, buildings and overhead, intervention delivery, training, and equipment. Time-motion observations following protocols previously used by our group in Mozambique [73] will estimate personnel time needed for EUC, SPI, and TCBT-S tasks.

### Economic evaluation

We will parameterize the model with costs and primary/secondary outcome data from the trial to simulate the health and economic impacts of scaling-up EUC, SPI alone, or TCBT-S in Mozambique at district, provincial, and national levels. Health outcomes include effects on suicidal behavior, suicidal ideation, and depression. The model will estimate the effects of scale-up scenarios on: (1) suicidal behavior; (2) suicidal ideation; (3) depressive symptoms; and (4) Disability Adjusted Life Years (DALYs) [74] averted; (5) cost per DALY averted; and (6) budgetary impact from the payer (Ministry/Mozambican Government) perspective. Different scenarios will examine rate and breadth of expansion (number/characteristics of future schools/districts/provinces). We will estimate incremental costs, as well as treatment costs incurred and averted due to SPI alone and TCBT-S. Throughout study implementation, all payer costs (MoH) will be collected and tabulated using activity-based cost menus to collect cost at start-up, implementation, and scale. Costs include but are not limited to: student visits; hospitalization; medication; personnel; supervision; supplies; buildings and overhead; intervention delivery; training; equipment; and any school resources utilized whether direct or in-kind. Time-motion observations following protocols previously used by our group in Mozambique [73] will estimate personnel time needed for EUC, SPI, and TCBT-S intervention implementation.

We will develop a Markov cohort model using R software reflecting the natural history of suicidal behavior, suicidal ideation, and depression progression and disability under different treatment scenarios. Similar Markov models have been used for previous cost-effectiveness analyses of suicide prevention and mental health interventions globally [75–84]. The model simulates a cohort of students stratified by types of suicidal ideation, behavior, death, and depression. The model will be parameterized with cost and outcome data from EUC/SPI/TCBT-S and school-level contextual data (location; size; type) and demographic data (grade; gender; age distributions). In line with previous studies and economic guidelines [74, 75], costs and effectiveness will be discounted at 3% annually (varied from 0–5% in sensitivity analyses). Disability weights for DALYs will be obtained from the Global Burden of Disease 2019 [85, 86]. Scale-up scenarios will model changes in rates of suicidal behavior, ideation, and depression based on study results and varied in sensitivity analyses. Prior to conducting cost-effectiveness analyses we will assess model validity/reliability by evaluating our model’s ability to accurately project trends in suicidal behavior.

We will project effects of SPI and TCBT-S on suicidal behavior, ideation, and depression compared to EUC. We will calculate the costs per suicidal behavior averted and the Incremental Cost-Effectiveness Ratio (ICER) per suicidal behavior averted and DALYs averted for each scale-up scenario. Following WHO guidelines [87–89], SPI alone or TCBT-S will be considered cost-effective if the ICER is less than Mozambique’s gross domestic product per capita. Analyses will be from the payer perspective, using economic productivity data to estimate disability averted and incorporating costs of healthcare assess. We will also estimate the budget impact, including costs incurred and averted, disaggregated by category to inform planning.

### Trial monitoring

An independent Data Safety and Monitoring Board (DSMB) will be convened for this study. The DSMB will be responsible for reviewing the trial protocol prior to participant enrollment to ensure that adequate patient safety protocols are in place. The DSMB will consist of experts in suicide prevention, the local Mozambican mental health context, and biostatistics. All will declare they have no conflicts of interest. They will also be responsible for assessing if study staff are responding appropriately to adverse events that arise during delivery of the intervention(s), whether related to study procedures or intervention services. The DSMB will also assess if the trial is on track to randomize and collect outcome data from an adequate number of participants answer the primary study question. They will review reported protocol violations and determine if the study treatment(s) or intervention(s) being delivered with adequate quality or fidelity to answer the primary study question. The Principal Investigator (BHW) will be primarily responsible for investigating adverse events (AEs) and serious adverse events (SAEs) to determine the potential relationship with study participation. In the event of the PI’s absence, investigations will be performed by study co-investigators. SAEs will be reported to the IRBs, DSMB, and NIMH within 72 h. If a suspension or change in protocol is recommended by the DSMB, the NIMH will be informed within 72 h. The DSMB or IRB report generating the concern and any other information to clarify the concern will be submitted to the IRBs, DSMB, and NIMH for review. The PI will coordinate the needed changes to allow the study protocols to be approved and the study approved to continue. The study will not proceed without approval of new protocols by the DSMB, IRBs and NIMH. No post-trial care is currently planned as part of this study.

### Dissemination

The project investigators are committed to the open and timely dissemination of research outcomes. Investigators recognize that results from this research are important for guiding policy and practice, and all investigators are aware of and agree to abide by the principles for sharing research resources, as described by NIH in ‘*Principles and Guidelines for Recipients of NIH Research Grants and Contracts on Obtaining and Disseminating Biomedical Research Programs.*’ The data generated in this research project will be presented at national or international conferences and published in a timely fashion. All final peer-reviewed manuscripts that arise from this proposal will be submitted to the digital archive PubMed Central.

To facilitate dissemination of knowledge gained from this research, every effort will be made to share findings in a manner conducive to support mental health training of primary care workers and mental health policy. Open access journals will be prioritized for publication. The inclusion of Ministry of Health staff on this team and collaboration with the Ministry of Health will ensure rapid translation of findings to policy. De-identified data will be retained at CSM-Mozambique to facilitate local use of findings and ongoing incorporation of research into improved practice and policy. Local dissemination of results will occur at target schools, be disseminated to participant students and families, be shared with the Ministries of Health and Education, and be presented at national Mozambican health conferences.

## Discussion

This study is innovative in simultaneously testing SPI as a brief intervention alone and SPI integrated into a transdiagnostic CBT-based therapy (TCBT-S) to inform implementation and scale-up to address the urgent need of youth suicide prevention in resource-limited contexts. This study will not only be the first – to our knowledge – to test these innovations in the Sub-Saharan African WHO region, but will also examine exploratory mechanisms of SPI and TCBT-S effects to inform the optimization of suicide prevention interventions. Mechanistic analyses will allow us to optimize the SPI and TCBT-S interventions in the future to maximally activate pathways of effects and match/tailor interventions to diverse barriers and contexts for suicide prevention interventions.

To date, nearly all studies of SPI interventions have been tested among high-income populations and specifically active-duty service members in the United States, veterans in VA-affiliated medical centers, or adults in emergency departments [63]. Ongoing questions linger regarding the effectiveness and the potential need to adapt SPI interventions for community settings versus more crisis-oriented settings [63]. Leaders in the field have advocated that future studies testing suicide prevention interventions specify intervention-mechanism-outcome linkages and test these mechanisms through causal pathway analyses [63]. Causal pathway analyses, and the specification of mechanisms of effect, are necessary to: (1) better understand why unsuccessful interventions for suicide prevention fail; (2) inform the development of more effective and efficient interventions; (3) identify mutable targets for future interventions; and (4) inform tailoring and matching interventions to barriers and contexts [90, 91]. Despite this urgent need in the field, to our knowledge only one ongoing study is explicitly examining mechanisms of SPI, and this study is being conducted in the United States among adults presenting in emergency rooms [60]. Previous mechanistic models of cognitive behavioral interventions for suicide prevention have similarly been conducted in high-income contexts and among active duty US army personnel [61, 62].

Understanding which components of an intervention are critical to effectiveness and which components may be adapted to suit context is critical to informing scale-up. The present study provides a unique opportunity to help distinguish the “hard core” of SPI and TCBT-S from their adaptable “soft periphery”. By pairing a rigorous three-arm cluster randomized trial with extensive implementation outcome data collection as well as exploratory analyses of mechanisms of intervention effects, this study builds from prior studies from high-income contexts and will help inform which intervention components are essential to effectiveness as researchers and implementers seek to scale-up SPI and cognitive behavioral interventions for suicide prevention globally. This project will also help generate an understanding of the overall “transferability” of the SPI and TCBT-S interventions, meaning the degree to which they can be adapted and tailored to meet the needs of contexts and populations of interest [39, 92].

In an environment where 90% of adolescents who die by suicide live in LMICs and countries like Mozambique have suicide rates almost three times the global average, this study will generate innovative and essential knowledge directly aimed at informing investments and scale-up of interventions to safeguard youth mental health. If effective, SPI or TCBT-S have a large potential to be rapidly scaled-up to save the lives of underserved adolescents globally.

## Supplementary Information

Supplementary Material 1.

## Data Availability

No datasets were generated or analysed during the current study.

## Abbreviations

AE: Adverse Events
C-SSRS: Columbia Suicide Severity Rating Scale
CBT: Cognitive Behavioral Therapy
CETA-MZ: Common Elements Treatment Approach – Mozambique
CFIR: Consolidated Framework for Implementation Research
CNBS: Mozambican National Committee of Bioethics in Health
CSM: Mozambique Health Committee
cRCT: Cluster Randomized Trial
DALY: Disability Adjusted Life Year
DSMB: Data Safety and Monitoring Board
EUC: Enhanced Usual Care
FGD: Focus Group Discussion
ICER: Incremental Cost-Effectiveness Ratio
IDI: In Depth Interview
LMIC: Low-and Middle-income Countries
NIH: National Institutes of Health
NIMH: National Institute of Mental Health
MoH: Ministry of Health
PHQ-9-MZ: Patients Health Questionnaire 9 Item for Mozambique
PI: Principal Investigator
RE-AIM: Reach, Effectiveness, Adoption, Implementation, and Maintenance Framework
SAE: Severe Adverse Event
SB: Suicidal Behavior
SEM: Structural Equation Model
SPI: Safety Planning Intervention
SPIRS-MZ: Safety Plan Intervention Rating Scale for Mozambique
TCBT-S: Transdiagnostic Cognitive Behavioral Therapy Intervention for Suicide Prevention
WHO: World Health Organization
